# Early Feed Restriction Programs Metabolic Disorders in Fattening Merino Lambs

**DOI:** 10.3390/ani8060083

**Published:** 2018-05-31

**Authors:** Javier Frutos, Sonia Andrés, Erminio Trevisi, David R. Yáñez-Ruiz, Secundino López, Alba Santos, F. Javier Giráldez

**Affiliations:** 1 Instituto de Ganadería de Montaña, CSIC-Universidad de León, Finca Marzanas s/n, 24346 Grulleros, León, Spain; jdefrutos@igm.csic.es (J.F.); s.lopez@unileon.es (S.L.); alba.santos@igm.csic.es (A.S.); j.giraldez@eae.csic.es (F.J.G.); 2 Faculty of Agriculture, Food and Environmental Science, Institute of Zootechnics, Università Cattolica del Sacro Cuore, Via Emilia Parmense 84, 29122 Piacenza, Italy; erminio.trevisi@unicatt.it; 3 Estación Experimental del Zaidín, CSIC, Profesor Albareda 1, 18008 Granada, Spain; david.yanez@eez.csic.es

**Keywords:** metabolic syndrome, nutritional programming, inflammation, ruminal acidosis, feed efficiency, microbiota

## Abstract

**Simple Summary:**

Inadequate nutrition of lambs during early life may compromise their health status during their whole lifetime. The aim of this study was to investigate the long-term effects of milk restriction during the suckling period on biochemical, immunological, hepatic, and ruminal parameters of fattening lambs. The results obtained reveal that early feed restriction during the suckling period of merino lambs promotes systemic metabolic disorders during the fattening phase that are not related to ruminal acidosis occurrence. This information may contribute to design strategies to enhance the health status of lambs undergoing milk restriction due to low milk production (e.g., udder problems) or lack of lamb vitality.

**Abstract:**

Early postnatal nutrition may have a significant subsequent impact on metabolic disorders during the entire lifespan of lambs. The aim of the present study was to describe the changes in biochemical, immunological, hepatic, and ruminal parameters of fattening lambs derived from early feed restriction during the suckling phase. Twenty-four merino lambs (average body weight, BW, 4.81 ± 0.256 kg) were used, 12 of them were milk-fed ad libitum (ADL) remaining permanently with their dams, whereas the other 12 lambs (restricted, RES) were subjected to milk restriction. After weaning, all the lambs were fed 35 g/kg BW per day of the same complete pelleted diet to ensure no differences between groups in dry matter intake (603 vs. 607 g/day for ADL and RES lambs, respectively, *p* = 0.703), and were slaughtered at a BW of 27 kg. Biochemical profiles revealed higher concentrations of ceruloplasmin and low-density lipoproteins, whereas insulin concentration was lower in the RES lambs compared to the ADL group. Liver thiobarbituric acid reactive substances were lower in the RES lambs. No significant differences in ruminal or blood immunological parameters were found. In conclusion, early feed restriction promoted metabolic disorders not related to ruminal acidosis occurrence, which can compromise the health status during the fattening period of merino lambs.

## 1. Introduction

Exposure to undernutrition at early stages of development programs the major components of the metabolic syndrome (e.g., altered cholesterol metabolism, atherogenic dyslipidemia, endothelial dysfunction), thus increasing the risk of cardiovascular disease in humans later in life [1]. This metabolic syndrome may also promote insulin resistance (and hence diabetes mellitus), visceral adiposity, obesity, and associated chronic low-grade inflammation with adverse long-term consequences on health status during the adult life [2]. Although there is information documenting these effects in humans, there are almost no studies describing the effects of early feed restriction on metabolic disorders in livestock species [3]. Filling this gap of knowledge is extremely important, since early feed restriction can take place during the suckling period under farm conditions for several reasons (e.g., reduction of milk production of the ewe or pathologies in the udder), which might compromise the health status of offspring later in life.

In ruminants, another important issue related to metabolic disorders is the microbiota firmly attached to the ruminal epithelium (epimural), since epithelial cells are fully competent for the recognition of microbial components and secretion factors modulating the inflammatory response [4,5]. Epimural microbiota may influence signaling pathways regulating the ruminal inflammatory response, such as cytokine production [6] or toll-like receptors (TLRs) in the membrane of several cell types (e.g., macrophages, T and B cells, or non-immune cells). Once established, the epimural bacterial community seems to be less influenced by the diet than microbiota associated to digesta [7,8,9]. Therefore, any factor (e.g., early feed restriction) modifying the composition of the epimural microbiota in the rumen might modulate the inflammatory response and hence the susceptibility of animals to the development of ruminal acidosis during the fattening phase, when non-optimal diet conditions are prevalent [6,8]. Decreasing the susceptibility to ruminal acidosis through the establishment of a healthy microbiota may improve animal welfare and performance. To our knowledge, there are no studies describing the effects of early feed restriction on ruminal epimural bacterial community and the subsequent implications for ruminal acidosis occurrence. Therefore, the effects of moderate early feed restriction on both systemic metabolic disorders and the ruminal status of fattening merino lambs after weaning were investigated in the current study.

## 2. Materials and Methods

The Animal Experimentation Committee of our research center (Instituto de Ganadería de Montaña (CSIC-Universidad de León)) authorized all of the handling practices included in the experimental design (protocol number 2015-04), which followed all the recommendations of the Directive 2010/63/EU of the European Parliament and of the Council on the protection of animals used for scientific purposes.

Twenty-four male lambs (merino breed) penned individually with their mother ewes during the suckling period were used in this experiment, as previously described by Santos et al. [10] and Frutos et al. [11]. The lambs were assigned randomly either to the ad libitum group (ADL, n = 12 lambs), in which each lamb remained all day (permanently) with the mother, or to the restricted group (RES, n = 12 lambs), where lambs were separated from the dams from 09:00 to 18:00 h every day. Dams of the RES group were injected with oxytocin to remove alveolar milk and then milked at 17:00 h before joining the lambs until the next day. Each lamb was weaned progressively starting at 13.5 kg body weight (BW). Weaned lambs weighing 15 kg of BW were penned individually, had free access to fresh drinking water, and were offered the same complete pelleted diet (CPD) at 35 g/kg BW per day throughout the fattening period to avoid differences in dry matter intake as explained elsewhere [10,12,13]. All the lambs received the CPD as a single daily meal at 09:00 h, and the amount of feed offered was adjusted twice a week based on the BW. Ingredients and chemical composition of CPD have been previously reported by Frutos et al. [11]. Finally, all the animals were slaughtered after a fattening period of at least 50 days when they reached the target BW of 27 kg, as explained previously by Santos et al. [10] and Frutos et al. [11].

### 2.1. Liver and Ruminal Parameters

A piece of liver was cut and kept at −80 °C until thiobarbituric acid reactive substances (TBARS) were determined [14]. Samples of ruminal wall tissue from two locations (posterior part of dorsal sac and anterior part of ventral sac) were rinsed three times with sterile phosphate-buffered saline (PBS, pH = 7.0) to remove the digesta. Each sample was divided into several portions and preserved for analysis of ruminal epimural microbiota (stored at −80 °C for 48 h, then freeze-dried), gene expression of cytokines and toll-like receptors (stored at −80 °C), immunoglobulin A (IgA) quantification (stored at −20 °C), and histological examination (fixed by immersion in 10% buffered formalin for one week). Ruminal epimural microbiota was assayed by DNA extraction and quantitative real-time PCR (qPCR) according to Andrés et al. [15] and Vargas et al. [16]. Gene expression of cytokines and toll-like receptors was performed by RNA extraction of rumen samples and real-time quantitative reverse transcription PCR (RNAlater Invitrogen, Lithuania) according to Frutos et al. [11]. IgA quantification in serum and ruminal mucosa was determined according to Ahmed et al. [17]. Color and histological analysis of ruminal samples were performed as described by Álvarez-Rodríguez et al. [18].

### 2.2. Blood Sampling, Biochemical Profile Analysis, and Flow Cytometry

All the animals had blood samples taken three times during the experiment (suckling, pre-weaning, and fattening period) early in the morning. Blood samples were taken by jugular venipuncture into tubes containing either no anticoagulant or lithium-heparin. Tubes with no anticoagulant were allowed to clot in a water bath at 37 °C for 30 min and then centrifuged at 3520× *g* for 16 min at 4 °C, and serum obtained was frozen at −80 °C until used to determine IgA. Lithium-heparin tubes were placed in iced water and centrifuged at 3520× *g* for 16 min at 5 °C. Plasma samples obtained were frozen at −80 °C until used for ensuing determinations, as described elsewhere [19]. Inflammatory response tests included positive acute-phase proteins (haptoglobin and ceruloplasmin) and negative acute-phase proteins (albumin, paraoxanase, and vitamin A, as index of its carrier retinol binding protein). Total bilirubin, aspartate aminotransferase (AST) and gamma glutamyl transpeptidase (GGT) were the liver profile parameters. Plasmatic indices of energy status were glucose, non-esterified fatty acids (NEFA), β-hydroxybutyrate, cholesterol, low-density lipoprotein (LDL), high-density lipoprotein (HDL), insulin, and triglycerides. The indicators of protein metabolism were urea, creatinine, total protein, and globulins. Assessment of oxidative stress and related variables included reactive oxygen metabolites (ROM), antioxidants (ferric reducing ability of plasma, FRAP), superoxide dismutase (SOD), vitamin E (α-tocopherol), and myeloperoxidase. Some minerals (Ca, Zn, Mg) were also determined in plasma. Haptoglobin, ceruloplasmin, albumin, paraoxanase, total bilirubin, AST, GGT, glucose, NEFA, β-hydroxybutyrate, total cholesterol, LDL, HDL, triglycerides, urea, creatinine, ROM, FRAP, SOD, myeloperoxidase, Ca, Zn, Mg, and total protein were determined by an autoanalyzer for biochemical chemistry ILAB 650 (Instrumentation Laboratory, Lexington, MA, USA). Globulin was calculated as the difference between total protein and albumin. Insulin, specific for sheep, was measured using an ELISA assay (Mercodia Ovine Insulin ELISA, Uppsala, Sweden) in microplates (Synergy 2, BioTek, Winooski, VT, USA). Plasma vitamins A and E were measured only during the fattening period by extraction with hexane and reverse-phase high-performance liquid chromatography using Spherisorb ODS-2, 3 m, in a 150 × 4.6 mm column (Alltech, Deerfield, IL, USA); an ultraviolet/visible detector set at 325 nm (for vitamin A) or 290 nm (for vitamin E); and 80:20 methanol:tetrahydrofurane as the mobile phase. Finally, during the fattening period, two more heparinized tubes were collected from each lamb in order to quantify lymphocyte population counts by flow cytometry according to Morán et al. [20].

### 2.3. Statistical Analysis

Data of liver TBARS values, color of ruminal papillae, histological measurements, plasma vitamins A and E, flow cytometry, IgA quantification, gene expression (TLRs and cytokines), and quantification of microbiota were analyzed by one-way ANOVA using the general linear model (GLM) procedure of SAS (SAS Institute Inc., Cary, NC, USA) with the dietary treatment (ADL or RES during the suckling period) as the only source of variation.

Data of biochemical parameters measured at several growth stages (suckling, pre-weaning, and fattening period) were analyzed as a repeated measures design using the MIXED procedure of SAS. Dietary treatment and growth stage were included in the model (the main effects of both factors and their interaction) as fixed effects. In all cases, the individual lamb was considered as the experimental unit and included in the model as a random effect. For each variable, the statistical model was fitted with competing covariance structures among the repeated measurements within a lamb (compound symmetric, unstructured, autoregressive, Toeplitz, and their heterogeneous versions) comparing the information criteria observed in each case. The covariance structure resulting in the smallest Akaike’s and Bayesian information criteria was selected. The Tukey–Kramer multiple-comparison test was used to determine the significance of treatment effects (ADL vs. RES) within each time point (sucking, pre-weaning, and fattening period). Significance was declared at *p* < 0.05 and a tendency at *p* < 0.10.

## 3. Results

### 3.1. Biochemical Profile

The effects of early feed restriction on biochemical profile during the suckling, pre-weaning, and fattening periods are presented in Table 1. Insulin concentration was lower in RES lambs (*p* = 0.034), but whereas there was a significant difference between ADL and RES lambs during the sucking period (579 vs. 258 ng/L), the difference was not significant (*p* > 0.05) for the fattening phase (254 vs. 269 ng/L). The concentration of SOD in plasma, a parameter related to oxidative stress, was similar for both groups during the suckling period (0.63 vs. 0.64 U/L for ADL and RES lambs, respectively), and was significantly (*p* < 0.05) greater in RES (1.24 U/L) than in ADL (0.89 U/L) lambs for the fattening phase. Total cholesterol (*p* = 0.020) and plasma lipoproteins (*p* = 0.046 for LDL and *p* = 0.039 for HDL) were higher in early feed-restricted compared with ADL lambs. Ceruloplasmin (one of the positive inflammatory indicators) concentration in plasma was significantly (*p* = 0.009) greater and total bilirubin was lower (*p* < 0.001) in RES compared with ADL lambs. The rest of the parameters related to energy (e.g., NEFA, β-hydroxybutyrate, and glucose), protein metabolism (e.g., creatinine, AST, and urea), or inflammation (e.g., haptoglobin and paraoxonase) were not affected (*p* > 0.05) by early feed restriction.

### 3.2. Immunological Parameters

No significant differences between experimental groups were observed in blood lymphocyte populations (flow cytometry) or IgA concentration in plasma and ruminal mucosa (Table 2). There were no significant effects of early feed restriction on mRNA expression of interferon gamma (IFN-γ), transforming growth factor β (TGF-β), or TLRs in ruminal mucosa (Table 3).

### 3.3. Liver Antioxidant Status (TBARS), Ruminal Characteristics, and Epimural Microbiota

The liver TBARS concentrations (inversely correlated to the antioxidant status) were significantly higher in ADL than in RES lambs (5.17 vs. 3.22 μg malonaldehyde (MDA)/g sample, RSD = 1.53, *p* = 0.005).

**Figure 1:**
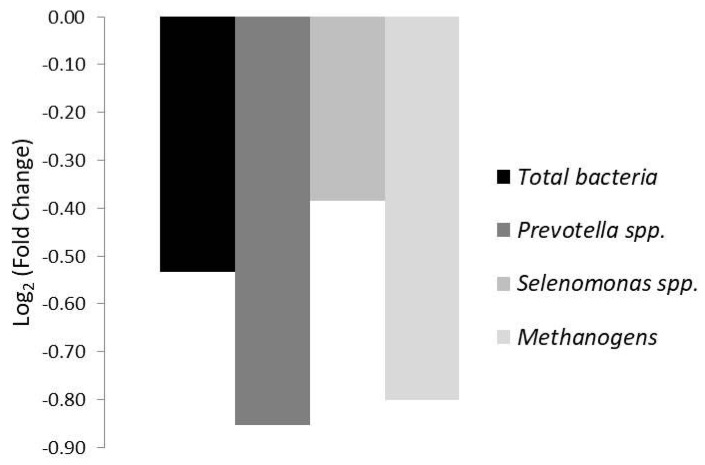
Relative quantitation compared to lambs fed ad libitum (ADL) of 16S rRNA copy numbers of microbial groups attached to the ruminal mucosa after early feed restriction (RES) during the suckling period. Fold-changes for specific amplicon groups were calculated as the log_2_ ratio of normalized abundances. No significant differences in copy number (*p* > 0.05, Tukey’s method of SAS) were observed between groups.

No significant differences were observed between treatments in color, height, or width of ruminal papillae, or thickness of stratum corneum (Table 2). The amount of total bacteria and the relative abundance (quantified by qPCR) of the microbial groups examined in the rumen (e.g., *Prevotella* spp., *Selenomonas ruminantium*, and methanogens) was not significantly affected by treatment (Figure 1).

## 4. Discussion

Early feed restriction can program metabolic risk and the development of pathological processes later in life [2,21], and can also influence the colonization of ruminal mucosa by commensal bacteria and, consequently, the inflammatory response [22]. Therefore, the possible consequences of moderate early feed restriction in suckling lambs for the occurrence of systemic and/or ruminal metabolic disorders were investigated comprehensively in this study.

Inadequate nutrient intake leads to a condition of negative energy balance and mobilization of lipids, thus increasing NEFA concentration and suppressing important immune functions [23,24]. However, the lack of differences in NEFA and immunity parameters such as blood lymphocytes (flow cytometry) or serum IgA suggest that RES lambs did not suffer a severe undernutrition or an adverse effect on their immune system. The moderate early feed restriction undergone by the RES lambs impaired β-oxidation of fatty acids [12] and promoted fat accumulation in the liver, carcass, and intramuscular depots [10,13], which would explain the lower liver TBARS values observed in these animals.

Early moderate feed restriction reduces insulin secretion and modifies insulin sensitivity in a tissue-specific manner in order to ensure enough glucose uptake by fundamental organs such as brain and heart during early life [2,21,25]. This adaptation mechanism during the feed-restriction period, together with the lower levels of bilirubin [26], might have induced insulin resistance so that more insulin (not detected probably because sampling was early in the morning before delivery of CPD) would be required to metabolize glucose during the fattening phase of RES lambs. Insulin is a lipogenic hormone, explaining the greater fat accumulation observed in the RES group [10,13]. Therefore, the observations of the present study support a metabolic syndrome induced in the RES lambs by early feed restriction, which might have been at least partly responsible for the lower feed efficiency traits in this group of lambs, as previously published by Santos et al. [13].

In line with the previous statement, the higher levels of ceruloplasmin (a positive acute-phase protein) observed for the RES lambs suggest a possible detrimental effect on health/metabolic status of these animals during the fattening period. This parameter, together with the lower levels of total bilirubin (with antioxidant properties; [27]) and higher values of SOD (with ROM reduction capability and anti-inflammatory properties), suggest a persisting inflammatory condition promoted by early feed restriction in RES lambs [23], as previously reported in the ileum [11]. This seems to be corroborated by the significantly higher cholesterol levels observed in the RES lambs when compared to the ADL group (probably enhanced by the lower plasmatic bilirubin concentration of RES lambs [26]), because the higher cholesterol concentration may promote inflammatory responses in different species [28]. The activity of enzymes involved in cholesterol synthesis and excretion may be programmed during early life (e.g., by feed restriction), thus affecting lipid metabolism and inflammatory profile later in life [1,2].

The lack of changes in the total bacteria or other microbial groups attached to the ruminal epithelium indicates no impact of moderate early feed restriction on the colonization of ruminal mucosa [29]. This circumstance, together with the lack of differences in ruminal fermentation parameters [13] and the identical morphometric and color parameters of the ruminal mucosa, suggest no differences in ruminal acidosis incidence promoted by early feed restriction despite of the differences in cholesterol [30]. The absence of significant differences in the gene expression of both TLRs (key inducers of inflammation probably through NF-κB activation) and cytokines in the ruminal wall also seems to corroborate this situation.

## 5. Conclusions

Moderate early feed restriction during the suckling period of merino lambs promotes metabolic alterations during the fattening phase, mainly related to lipid metabolism and adiposity. This event, which might impair health status, is independent of ruminal acidosis occurrence during the fattening period.

## Figures and Tables

**Table 1:** Biochemical parameters of fattening lambs fed ad libitum (ADL) or restricted (RES) diets during the suckling period.

	Treatment		Growth Stage			SED_1_	SED_2_	*p*-Values		
	ADL	RES	SCK	PWN	FTT			T	GS	T*GS
Haptoglobin (g/L)	0.22	0.23	0.21 ^A^	0.20 ^A^	0.27 ^B^	0.022	0.025	0.886	0.016	0.470
Ceruloplasmin (µmol/L)	2.34 ^A^	3.10 ^B^	2.81	2.80	2.55	0.262	0.222	0.009	0.427	0.308
β-hydroxybutyrate (mmol/L)	0.29	0.31	0.30	0.27	0.32	0.027	0.033	0.495	0.393	0.316
Paraoxonase (U/L)	117	125	88 ^A^	150 ^C^	125 ^B^	15.0	7.63	0.624	<0.001	0.018
ROM (mg/100 mL)	16.5	17.6	17.0	17.9	16.2	1.22	1.04	0.382	0.276	0.020
Myeloperoxidase (U/L)	434	466	400 ^A^	417 ^A^	533 ^B^	52.1	49.8	0.554	0.021	0.848
FRAP (µmol/L)	154	154	147	152	164	10.3	12.6	0.945	0.400	0.207
SOD (U/L)	0.69 ^A^	1.04 ^B^	0.64 ^A^	0.89 ^B^	1.07 ^B^	0.077	0.094	<0.001	<0.001	0.043
Ca (mmol/L)	3.02	2.96	3.16 ^B^	3.09 ^B^	2.73 ^A^	0.044	0.043	0.174	<0.001	0.200
Mg (mmol/L)	0.92	0.90	0.86 ^A^	0.87 ^A^	0.99 ^B^	0.022	0.016	0.557	<0.001	0.461
Zn (mmol/L)	15.8	16.5	18.9 ^C^	16.0 ^B^	13.6 ^A^	0.959	1.174	0.481	<0.001	0.196
Retinol (µg/100 mL)	40.2	39.9				3.43		0.935		
Tocopherol (µg/mL)	0.23	0.23				0.016		0.867		
AST (U/L)	146	162	73 ^A^	87 ^A^	301 ^B^	36.13	44.25	0.656	<0.001	0.763
GGT (U/L)	126	102	210 ^B^	69 ^A^	63 ^A^	22.91	27.12	0.298	<0.001	0.271
Total bilirubin (µmol/L)	2.10 ^B^	0.93 ^A^	2.73 ^B^	1.06 ^A^	0.76 ^A^	0.314	0.3770	<0.001	<0.001	0.661
Creatinine (µmol/L)	63.5	63.7	57.5 ^A^	65.2 ^B^	68.1 ^C^	1.73	1.113	0.937	<0.001	0.385
Glucose (mmol/L)	6.66	6.31	7.47 ^B^	6.76 ^B^	5.22 ^A^	0.212	0.164	0.109	<0.001	0.675
Insulin (ng/L)	497 ^B^	289 ^A^	418 ^B^	499 ^B^	262 ^A^	92.2	106.8	0.034	0.008	0.025
Urea (mmol/L)	6.89	7.45	4.59 ^A^	6.09 ^B^	10.84 ^C^	0.37	0.36	0.143	<0.001	0.009
Protein (g/L)	63.3	63.3	62.9	63.3	63.7	1.29	1.21	0.978	0.816	0.423
Albumin (g/L)	34.8	34.7	33.4 ^A^	35.5 ^B^	35.3 ^B^	0.41	0.42	0.947	<0.001	0.706
Globulin (g/L)	28.6	28.6	29.5	27.8	28.4	1.23	1.37	0.995	0.440	0.420
Cholesterol (mmol/L)	2.06 ^A^	2.50 ^B^	3.08 ^B^	2.72 ^B^	1.05 ^A^	0.173	0.193	0.020	<0.001	0.497
LDL (mmol/L)	0.56 ^A^	0.72 ^B^	0.88 ^C^	0.73 ^B^	0.32 ^A^	0.076	0.750	0.046	<0.001	0.746
HDL (mmol/L)	1.10 ^A^	1.33 ^B^	1.67 ^B^	1.50 ^B^	0.48 ^A^	0.103	0.107	0.039	<0.001	0.392
NEFA (g/L)	0.43	0.44	0.63 ^C^	0.53 ^B^	0.14 ^A^	0.042	0.44	0.755	<0.001	0.639
Triglycerides (mmol/L)	0.53	0.54	0.75 ^B^	0.68 ^B^	0.18 ^A^	0.060	0.072	0.872	<0.001	0.977

^A, B, C^ Values within a row with different superscripts differ significantly at *p* < 0.05 for dietary treatment or growth stage. AST, aspartate transaminase; FRAP, ferric reducing ability of plasma; FTT, fattening period; GGT, gamma-glutamyltransferase; GS, growth stage; HDL, high-density lipoproteins; LDL, low-density lipoproteins; NEFA, non-esterified fatty acids; PW, pre-weaning period; ROM, reactive oxygen metabolites; SCK, suckling period; SED_1_, standard error of the difference to compare dietary treatments; SED_2_, standard error of the difference to compare growth stage; SOD, superoxide dismutase; T, dietary treatment; T*GS, interaction between dietary treatment and growth stage.

**Table 2:** Lymphocyte counts measured by flow cytometry in peripheral blood, ruminal parameters, and immunoglobulin A (IgA) concentration in serum and ruminal mucosa of fattening lambs being fed ad libitum (ADL) or restricted (RES) diets during the suckling period.

	ADL	RES	RSD	*p*-Value
**Lymphocytes (%) ^1^**				
CD4^+^	9.29	12.2	7.4	0.351
CD8^+^	4.83	5.26	3.69	0.779
CD45^+^	17.5	19.5	10.4	0.649
CD21^+^	21.8	21.9	14.0	0.719
CD4^+^/CD8^+^	1.79	2.32	1.51	0.403
**Ruminal parameters**				
Mean gray value ^2^	130	126	8.5	0.297
Papillae length AV (µm)	3985	3649	856	0.359
Papillae length PD (µm)	3878	3874	893	0.993
Papillae width AV (µm)	1244	1178	177	0.383
Papillae width PD (µm)	1289	1318	220	0.771
*Stratum corneum* thickness AV (µm)	61.3	59.1	13.6	0.690
*Stratum corneum* thickness PD (µm)	60.2	70.6	19.8	0.208
**IgA**				
Blood (pg/mL)	7921	8981	2595	0.373
Rumen (pg/µg total protein)	5.87	4.51	3.52	0.413

^1^ Percentage of positive stained cells in sample populations of 10,000 individual cells. ^2^ Colour of ruminal mucosa, from a scale where 0 is black and 256 white. AV, anterior part of ventral sac; MDA, malonaldehyde; PD, posterior part of dorsal sac; RSD, residual standard deviation.

**Table 3:** Cytokines and toll-like receptors (TLRs) mRNA expression in ruminal mucosa of fattening lambs being fed ad libitum (ADL) or restricted (RES) diets during the suckling period.

	ADL	RES	RSD	*p*-Value
**Cytokines (ΔCq) ^1^**				
IFN-γ	11.3	11.2	1.6	0.927
TGF-β	6.32	6.52	0.65	0.558
**TLR (ΔCq) ^1^**				
TLR_1_	11.3	11.9	0.8	0.072
TLR_2_	23.6	23.5	1.9	0.903
TLR_3_	14.8	15.4	0.8	0.056
TLR_4_	12.2	12.4	0.7	0.521
TLR_5_	23.7	23.6	1.7	0.870
TLR_6_	11.7	12.1	1.0	0.364
TLR_7_	15.2	15.7	0.9	0.212
TLR_8_	18.0	17.8	0.8	0.577
TLR_9_	16.9	17.0	1.4	0.957
TLR_10_	14.0	13.9	1.3	0.938

^1^ Cq, quantification cycle. ΔCq = Cq (cytokines or TLRs) − Cq (β-actin). Lower Cq represents higher mRNA abundance level. IFN-γ, interferon gamma; RSD, residual standard deviation; TGF-β, transforming growth factor beta.
